# Clinical pattern and spectrum of endometrial pathologies in patients with abnormal uterine bleeding in Pakistan: need to adopt a more conservative approach to treatment

**DOI:** 10.1186/s12905-014-0132-7

**Published:** 2014-11-05

**Authors:** Mariam Abid, Atif Ali Hashmi, Babar Malik, Saroona Haroon, Naveen Faridi, Muhammad Muzzammil Edhi, Mehmood Khan

**Affiliations:** Department of Pathology and Microbiology, Aga Khan University Hospital, Karachi, Pakistan; Department of Histopathology, Liaquat National Hospital and Medical College, Karachi, Pakistan; Department of Medical Oncology, Sindh Institute of Urology & Transplantation, Karachi, Pakistan; Intern, Liaquat National Hospital and Medical College, Karachi, Pakistan; Dhaka Medical College, Dhaka, Bangladesh

**Keywords:** Endometrial pathology, Abnormal uterine bleeding, Endometrial biopsy, Endometrial polyp, Endometrial carcinoma, Endometrial hyperplasia

## Abstract

**Background:**

Abnormal uterine bleeding (AUB) is one of the most common debilitating menstrual problems and has remained one of the most frequent indications for hysterectomy in developing countries. Approximately in 40% of hysterectomy specimens, no definite organic pathology could be established. The problem is common worldwide but causes may vary from one region to another. This study may help gynecologists in our population to improve their therapeutic strategies by promoting minimally invasive uterus sparing modalities such as endometrial ablation and hysteroscopic resection of early proliferative lesions.

**Methods:**

It was a prospective, cross-sectional study conducted at Liaquat National Hospital from 15^th^ January 2010 till 14^th^ July 2011 over a period of 18 months. Women who underwent dilatation and curettage for endometrial sampling with complaints of AUB were included in the study and histopathologic spectrum was determined.

**Results:**

Polymenorrhea was the most common presenting pattern (30%, 72/241) with reproductive age women being the most susceptible (49.3%,119/241). The commonest histopathological spectrum was normal menstrual pattern (34%, 82/241) and the commonest pathology was hormonal imbalance (27%, 65/241), followed by endometrial polyp (14%, 34/241), chronic endometritis (12%, 28/241), atrophic endometrium (6%, 15/241), endometrial hyperplasia (5%, 12/241), and endometrial carcinoma (2%, 5/241). Chronic endometritis was commonly seen in reproductive age (18%, 21/119); hormonal imbalance (45%, 35/77) and endometrial hyperplasia (6.5%, 5/77) in perimenopausal age; endometrial polyp (35.5%, 16/45) and endometrial carcinoma (9%, 4/45) in postmenopausal age.

**Conclusion:**

Frequency of benign endometrial pathology is quite high in AUB, 236 participants (98%, 236/241). Histopathological spectrum in patients with AUB is quite variable with respect to age. The most common pattern of AUB was polymenorrhea. The most common pathology was hormonal imbalance. It is suggested that age was associated with more progressive lesions found in peri and postmenopausal age group such as endometrial hyperplasia and endometrial carcinoma. Yet endometrial polyp was the most common pathology found in postmenopausal women. Therefore, the management strategy should be individualized, as in most cases a restrictive approach is appropriate in order to avoid unnecessary hysterectomies.

## Background

Menstrual problems account for much of the morbidity, affecting one in every five women during their life span. Specifically, abnormal uterine bleeding (AUB) is one of the most common debilitating menstrual problems. A study based on epidemiology of menstrual disorders in developing countries revealed that the prevalence of AUB in developing countries including Pakistan was about 5-15% [1]. Within the study period there were 400 AUB cases out of 1600 gynaecological cases which gave an AUB prevalence of 25% per 100 gynaecological cases. Out of 400 AUB cases, 241 (60%) participants were recruited into the study. Risk factors of AUB include female genital tract pathologies, pregnancy related disorders, and systemic illnesses [2].

Spectrum of common pathologies that can be detected histologically in AUB include hormonal imbalance pattern (disorderly proliferative endometrium, non secretory endometrium with stromal and glandular breakdown, luteal phase defect and pill effect) atrophic endometrium, endometritis, endometrial polyp, endometrial hyperplasia and endometrial carcinoma. However endometrial pathologies were noted in only about half of the cases of AUB and hormonal imbalance pattern dominated the clinical picture [3,4].

AUB has remained one of the most frequent indications for hysterectomy in developing countries but 40% of cases were not associated with any definitive organic pathology [5]. Hysterectomy is often correlated with complications such as bleeding, bladder or bowel damage, infection, thrombosis, ovary failure and early onset of menopause [6]. Prevalence of abnormal uterine bleeding in developing countries including Pakistan is about 5-15% [1].One half of women by age 45.5 years, three quarter by age 47.8 years & 95% by age 50.8 years experience menstrual disturbance [1]. It has been estimated that around 6% of women aged 25–44 years consult their general physician about excessive menstrual loss every year, around 35% of these referred to hospital and 60% of them underwent hysterectomy in the next 5 years. Over 75000 hysterectomies are now carried out every year & 25-30% of them are for menstrual disturbance [7].

Hysterectomy is associated with high rate of morbidity (Ureteral injuries are common, owing to the size and location of the ureter, Bladder injuries occur in up to 2% of cases, hemorrhage occurs in 1% to 3% of patients, atelectasis, fallopian tube prolapse, thromboembolic disease, myocardial infarction, stroke, and renal failure) [3] and is employed as last prerogative in management of AUB in developed countries due to availability of minimally invasive surgical (MIS) modalities such as endometrial ablation and thermal balloon therapy [6,8,9]. MIS is equated with faster recovery, less pain, smaller incisions and shorter hospital stays. Hysteroscopic resection has proved invaluable in treating atypical proliferative lesions of endometrium as an alternative to hysterectomy [10].

Since endometrium is the best accessible tissue for histopathological evaluation of uterine bleeding, several methods are used for endometrial sampling among which Dilatation and Curettage is considered to be a method of choice [11] therefore used as standard practice in our set up. The aim of the present study was to determine the clinical spectrum and frequency of pathologies in endometrial biopsy of patients with AUB in our population.

## Methods

It was a prospective cross-sectional study conducted at Liaquat National Hospital (LNH) from 15^th ^January 2010 till 14^th^ July 2011. Ethics committee of Liaquat National hospital approved the study. LNH is a private sector tertiary care hospital serving the society for the last 56 years having approximately 700 beds with 30 medical and surgical specialties providing both inpatient and outpatient services. The sampling technique was consecutive non-probability purposive. Women who underwent dilatation and curettage (D & C) for endometrial sampling with complaints of AUB were included in the study and standard operating procedures were followed for obtaining histopathological sample [12]. All procedures were carried out by 3 different gynaecologists with more than 3 year post fellowship experience. The gynaecologists were blinded to the study objective. 241 endometrial biopsies fulfilling the inclusion criteria were included. Age, duration of abnormal uterine bleeding and observed histopathological spectrums were recorded on proforma and standard operating procedure were followed in specimen handling. Patients with systemic illness/cause of bleeding like low platelet count or miscarriage were excluded from the study. Informed consent was taken prior to procedure and an approval from institutional ethical review committee was taken antecedent to conducting the study. Cases were stratified into reproductive, peri-menopausal and post-menopausal age groups. Reproductive age was considered from puberty to perimenopausal age (approx. 12 to 39 years). Perimenopausal age was around menopause, and was different for every woman (approx. 40–50 years). Postmenopausal age group was defined as permanent cessation of menstruation, confirmed after 12 consecutive months of amenorrhea (average age 50 years onwards).

Specimens were immediately put in 10% formalin, appropriately labeled for patients name, gender, age and type of procedure/specimen and transferred to histopathology laboratory where a specific ID number was given to each specimen. Grossing of specimens was done using standard protocols and measurements were recorded. Tissue processing was performed and slides were stained with hematoxylin and eosin stains under strict quality assurance. Microscopic evaluation was done by two consultant histopathologists having post-fellowship experience of more than 5 years, blinded to the objectives of the study. Each sample was evaluated independently by the two consultant histopathologists when required. Data was entered into SPSS version 10. Results were presented as mean ± SD for age, frequencies and percentages was computed for descriptive variables i.e. normal menstrual pattern, hormonal imbalance pattern, endometrial polyp, chronic endometritis, atrophic endometrium, endometrial hyperplasia and endometrial carcinoma. Stratification of age and duration of abnormal uterine bleeding was done to control the confounding factor. Sample size was 241, calculated by taking endometrial carcinoma as the least prevalent pathology (p= 6%, d= 5%, confidence level as 95%).

### Definitions [11]

#### Menorrhagia

Prolonged (>7 days) or excessive (>80 mL daily) uterine bleeding occurring at regular intervals.

#### Metrorrhagia

Uterine bleeding occurring at irregular and more frequent than normal intervals.

#### Menometrorrhagia

Prolonged or excessive uterine bleeding occurring at irregular and more frequent than normal intervals.

#### Intermenstrual\irregular bleeding

Uterine bleeding of variable amounts occurring between regular menstrual periods.

#### Polymenorrhea

Uterine bleeding occurring at regular intervals of less than 21 days.

#### Oligomenorrhea

Uterine bleeding occurring at intervals of 35 days to 6 months.

#### Post menopausal bleeding

Bleeding after physiologic cessation (12 months or more) of menstruation.

## Results

Within the study period there were 400 AUB cases out of 1600 gynaecological cases which gave an AUB prevalence of 25% per 100 gynaecological cases. Out of 400 AUB cases, 241 (60%) participants were recruited into the study. Patients’ mean age was 40.3 years ±11.06 (18 – 75 years).Reproductive age group was the most common age group, 119 participants (49.3%, 119/241) presented with AUB, followed by peri-menopausal, 77 participants (32%, 77/241) and post menopausal age group, 45 participants (18.7%, 45/241).

175 participants were married (72.6%, 175/241) and 66 were unmarried (27.3%, 66/241).

**Figure 1:**
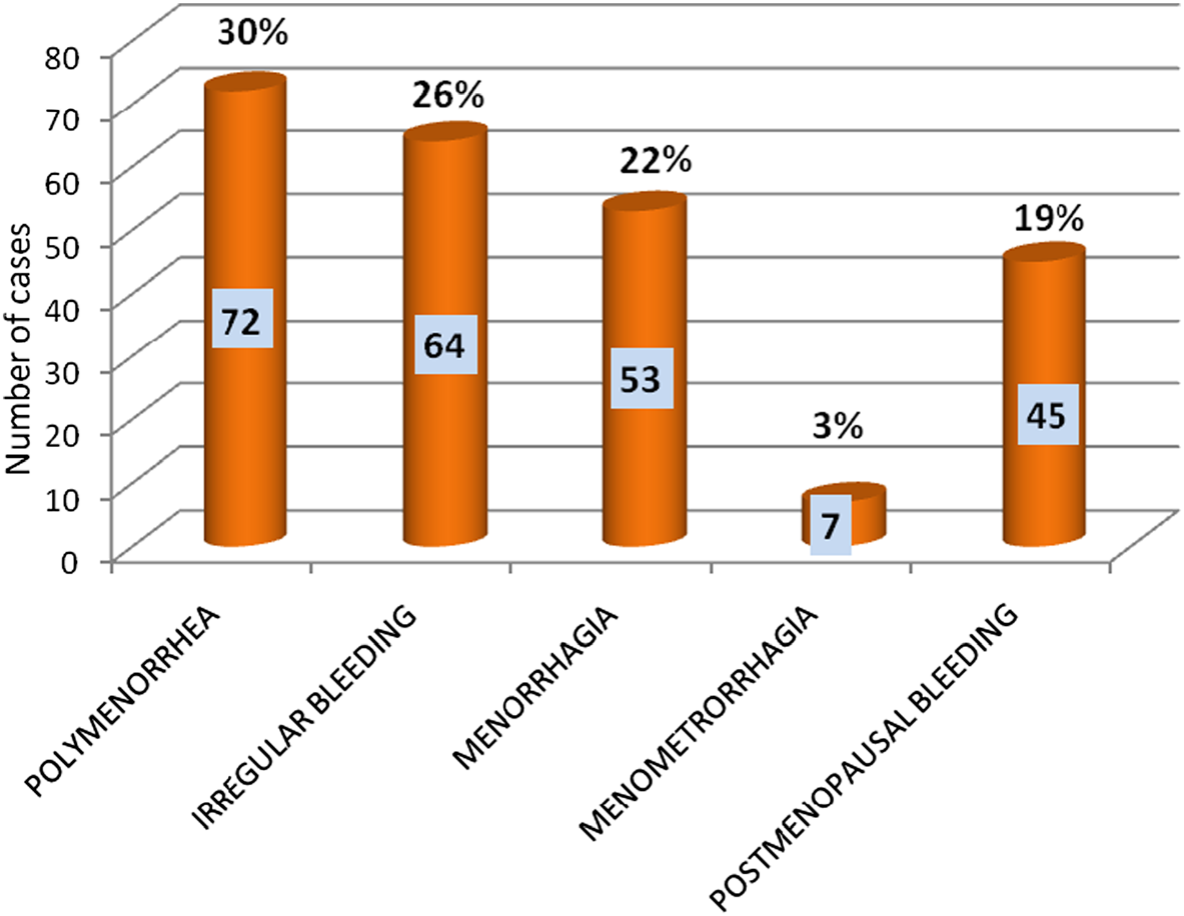
**Clinical pattern of abnormal uterine bleeding n= 241.**

**Figure 2:**
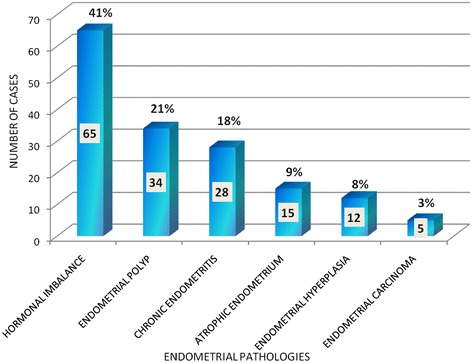
**Frequency of endometrial pathologies n= 159.**

72 participants (30%, 72/241) presented with polymenorrhea which was the most common pattern followed by irregular bleeding (26%, 64/241) (Figure 1). Major histological patterns observed were as follows, normal menstrual pattern in 82 participants (34%, 82/241), hormonal imbalance pattern in 65 (27%, 65/241), endometrial polyp in 34 (14%, 34/241), chronic endometritis in 28 (12%, 28/241), atrophic endometrium 15 (6%, 15/241), endometrial hyperplasia in 12 (5%, 12/241) and endometrial carcinoma was noted in 5 (2%, 5/241) participants (Table 1). Therefore endometrial pathologies were observed in 159 (66%, 159/241) participants (Figure 2), whereas normal menstrual pattern was seen in 82 (34%, 82/241) participants, of AUB.

**Table 1:** **Histopathological spectrum in patients with abnormal uterine bleeding**

**Histopathological spectrum**	**n (%)**
Normal menstrual pattern	82 (34%)
Hormonal imbalance	65 (27%)
Endometrial polyp	34 (14%)
Chronic endometritis	28 (12%)
Atrophic endometrium	15 (6%)
Endometrial hyperplasia	12 (5%)
Endometrial carcinoma	05 (2%)
**Total**	241 (100%)

Among 82 normal menstrual pattern, secretory endometrium was the most common pattern i.e. 49 (60%, 49/82) participants, followed by proliferative pattern endometrium 33 (40%, 33/82) participants. Hormonal imbalance pattern, chronic endometritis, endometrial hyperplasia and endometrial carcinoma was further stratified for detailed analysis. Among 64 cases of hormonal imbalance pattern, 52 participants, (81%, 52/64) were estrogen related and 12 participants (19%, 12/52) were progesterone associated. Among 28 cases of chronic endometritis, non-specific endometritis was observed in 27 cases (96.4%, 27/28), whereas only 1 (3.6%, 1/28) participants showed chronic granulomatous inflammation. Among 12 participants of endometrial hyperplasia, 7 participants (58%, 7/12) showed atypia whereas 5 participants (42%, 5/12) were without atypia. Among 5 participants of endometrial carcinoma, type I endometrial carcinoma was noted in 4 participants (80%, 4/5) while type 2 endometrial carcinoma was noted in 1 participants.

Stratification of age was done and frequency of endometrial pathologies was observed in different age groups (Table 2). In reproductive and peri-menopausal age groups hormonal imbalance pattern followed by endometrial polyp dominated the pathologic picture while in postmenopausal age group, endometrial polyp was the most frequent pathology (35.5%, 16/45) followed by atrophic endometrium (33.3%, 15/45). On the other hand the frequency of endometrial hyperplasia and carcinoma was quite low, 13.2%(6/45) and 9% (4/45) respectively (Table 2).

**Table 2:** **Histopathological spectrum in reproductive, peri and postmenopausal age groups**

**Endometrial pathologies**	**Reproductive age group**	**Perimenopausal age group**	**Postmenopausal age group**
Hormonal imbalance	26 (22%)	35 (45.5%)	4 (9%)
Endometrial polyp	10 (8.4%)	8 (10.4%)	16 (35.5%)
Chronic endometritis	21 (18%)	7 (9.1%)	0 (0%)
Atrophic endometrium	0 (0%)	0 (0%)	15 (33.3%)
Endometrial hyperplasia	1 (1%)	5 (6.5%)	6 (13.2%)
Endometrial carcinoma	0 (0%)	1 (1.2%)	4 (9%)
Normal menstrual pattern	61 (51%)	21 (27.3%)	0 (0%)
Total (241)	119	77	45

Thus in all 241 participants 236 (98%, 236/241) had benign histopathological spectrum; whereas malignancy was observed in only 5 participants (2%, 5/241).

## Discussion

Uterine bleeding presents as the most common symptom that confronts gynecologists, and poses a considerable health risk. AUB is excessive, erratic, or irregular bleeding usually associated either with hormonal disturbance or intrauterine pathology. It has been estimated that around 6% of women aged 25–44 years consult their general physician due to excessive menstrual loss every year. One of the major causes of excessive or erratic menstruation is inadvertent use of contraceptive modalities. In the past, repeated childbirth and lactation caused prolonged amenorrhea, thus alleviating proliferative activity of endometrium. AUB is of concern because it may have serious medical and social consequences, as bleeding may cause anemia, undue disruption of women’s daily activities and sexual life.

Endometrial assessment is performed to diagnose malignancy or pre-malignant conditions and to evaluate the hormonal influences of the endometrium. It is important to evaluate the endometrial histopathology in a woman who has no improvement in her bleeding pattern following a course of medical therapy of three months [12]. It is well established that AUB is significant cause of morbidity, but the underlying causes of AUB may vary from one region to another.

A total of 241 endometrial samples of patients with abnormal uterine bleeding were assessed. Maximum frequency of AUB was observed in reproductive (18-39 years) age group (49.3%, 119/241) followed by perimeopausal (40-50 years) (32%, 77/241) and postmenopausal (51 above) age groups (18.7%, 45/241). We observed that age was as an important factor governing the histological progression. Age was directly associated with increasing aggressiveness of lesions since more progressive lesions were found in peri and postmenopausal age group as compared to reproductive age group.

Abnormal uterine bleeding may present with variable patterns. Our study showed 30% (72/241) participants presented with polymenorrhea, 22% (53/241) with menorrhagia, 26% (64/241) with irregular bleeding, 3% (7/241) with menometrorrhagea and 19% (45/241) with post-menopausal bleeding.

We observed 34% (82/241) participants with normal menstrual pattern of endometrium while endometrial pathologies were noted in 66% (159/241). Among 82 cases of normal pattern endometrium, secretory pattern was seen in 60% (49/82) participants and proliferative pattern in 40% (33/82).

Among 159 cases of endometrial pathologies, hormonal imbalance pattern was the most frequent pathology (41%, 65/159). The histological spectrum observed in hormonal imbalance pattern among 65 cases was estrogen related pattern in 80% (52/65) and progesterone related pattern in 20% (13/65). Hormonal imbalance pattern was seen most commonly in perimenopausal age group (54%, 35/64). This correlates with the fact that perimenopausal age is a transition from normal ovulation to anovulation.

Endometrial polyp was observed in 14% cases (34/241), whereas among endometrial pathologies its frequency was found to be 21%, 34/159. We observed an increase in frequency of polyp with increasing age i.e. 8.4% in reproductive age group, 10.4% in perimenopausal age group and 35.5% in postmenopausal age group. Although polyp was the second most common pathology after hormonal imbalance pattern, nonetheless it is not regarded as a major risk factor for the development of carcinoma. Furthermore, endometrial polyp was the most common pathology in postmenopausal women in our study. None of the polyps showed atypical changes suggestive of development of carcinoma. Similar trends were observed in national studies [12,13]. Azim et al. demonstrated an increased frequency of polyp with advancing age 5%, 8% and 11% in reproductive, perimenopausal and postmenopausal age groups respectively [14]. Dresiler et al. also observed the same findings in Danish population [15].

Among 28 cases of chronic endometritis 97% (27/28) showed nonspecific endometritis and only 3% (1/28) showed chronic granulomatous inflammation. This entity was most commonly seen in reproductive and perimenopausal age group, since these age groups have a greater chance of exposure to caesarian sections, spontaneous and therapeutic abortion and intrauterine contraceptive devices etc. hence, prone to develop chronic endometritis. Some variation has been observed among national studies, Muzaffar et al. at Rawalpindi observed chronic endometritis in 13% [16], whereas Perveen et al. at Karachi in 37% participants [17]. The variation may be due to socioeconomic status, hygienic conditions or exposure to surgical intervention. On the other hand, since chronic endometritis may be due to some underlying pathology such as retained products of conception etc. a higher frequency of non-specific chronic endometritis in these studies may represent an uncovered underlying primary pathology. Meticulous attention to the endometrial sampling technique is needed in such circumstances.

We observed atrophic endometrium in 6% (15/241) and all patients were in the post menopausal age group, whereas reproductive and perimenopausal age group showed no such pattern. Anovulation seen in perimenopausal age group eventually leads to permanent loss of ovarian function manifested as atrophic or inactive endometrium. Among postmenopausal age group atrophic endometrium was found to be the second most common pathology (33%, 15/45).

Endometrial hyperplasia is the precursor of carcinoma and usually presents with abnormal uterine bleeding. We observed endometrial hyperplasia in 5% (12/241) participants, out of those 54% (7/12) showed hyperplasia with atypia and 46% (5/12) without atypia. It was seen most frequently in postmenopausal age group (46%) followed by perimenopausal (38.5%) and reproductive age group (15.5%) respectively. Perveen et al., Muzzafer et al. and Luqman et al. reported (96, 12%), (101, 24.7%) and (103, 14%) respectively [16-18]. Variation in population characteristics and use of hormonal therapy may also be the contributory factors to these differences, as use of estrogen therapy declined after its role in the development of endometrial carcinoma was uncovered. Varying incidence of endometrial hyperplasia was also evident in international literature. Behnamfar et al. evaluated cystic and adenomatous hyperplasia in addition to other patterns and reported an incidence of 9% and 10.9% respectively [15]. On the other hand, Dexus and Jyotsana reported higher frequency of endometrial hyperplasia, 21% and 22.6% respectively [13,19].

The frequency of endometrial carcinoma was low in our study (2%, 5/241). Type 1 endometrial carcinoma was seen in majority (80%, 4/5) while Type 2 in only 1 participant which is considered to be more aggressive (20%, 1/5). It was seen most commonly in post menopausal age group (4, 80%) followed by perimenopausal (1, 20%). These finding are similar to various national studies, where most of the investigators found a frequency of endometrial carcinoma in less than 1% cases [11,17]. We observed endometrial carcinoma was most commonly seen in postmenopausal age group yet its frequency was only 9% among other pathologies in this particular age group which is slightly lower than the Nepalese regional study who observed endometrial carcinoma in 17.6% [20] and Chinese Feng Y observed even higher incidence of endometrial carcinoma of 50% in PMB cases respectively [21]. The lower incidence of endometrial carcinoma in our population can be associated with early childbearing and multiparity. Proliferative activity markedly declines during pregnancy explaining the lower incidence of hyperplastic and neoplastic lesions in our population.

Due to its descriptive nature, our study may not be complete in all aspects. However, this study would provide a data base with regard to the histopathological spectrums in abnormal uterine bleeding in our population. This data base may help gynaecologist to modify their treatment strategies. Furthermore, based on common endometrial pathologies in our population, health literacy, screening programme etc., can be constructed effectively.

## Conclusion

Abnormal uterine bleeding was common at Liaquat National Hospital, Karachi, Pakistan and the commonest presentation was polymenorrhea. Histopahologically, most of the cases were benign-hormonal imbalance and the endometrial polyps were the commonest pathology in the reproductive age women and the post-menopausal women respectively. Therefore, management of AUB in our environment should be individualized and more restrictive with a view to conserve the uterus. Although a thorough endometrial sampling is recommended in post-menopausal women due to focal nature of endometrial polyps.
